# Neuronal dark matter: the emerging role of microRNAs in neurodegeneration

**DOI:** 10.3389/fncel.2013.00178

**Published:** 2013-10-10

**Authors:** Emily F. Goodall, Paul R. Heath, Oliver Bandmann, Janine Kirby, Pamela J. Shaw

**Affiliations:** Department of Neuroscience, Sheffield Institute for Translational Neuroscience, University of SheffieldSheffield, UK

**Keywords:** microRNA, neurodegeneration, Alzheimer's disease, Parkinson's disease, amyotrophic lateral sclerosis, Huntington's disease

## Abstract

MicroRNAs (miRNAs) are small, abundant RNA molecules that constitute part of the cell's non-coding RNA “dark matter.” In recent years, the discovery of miRNAs has revolutionised the traditional view of gene expression and our understanding of miRNA biogenesis and function has expanded. Altered expression of miRNAs is increasingly recognized as a feature of many disease states, including neurodegeneration. Here, we review the emerging role for miRNA dysfunction in Alzheimer's disease, Parkinson's disease, amyotrophic lateral sclerosis (ALS) and Huntington's disease pathogenesis. We emphasize the complex nature of gene regulatory networks and the need for systematic studies, with larger sample cohorts than have so far been reported, to reveal the most important miRNA regulators in disease. Finally, miRNA diversity and their potential to target multiple pathways, offers novel clinical applications for miRNAs as biomarkers and therapeutic agents in neurodegenerative diseases.

## Introduction

Arguably one of the most important discoveries in molecular biology in recent years has been the finding and characterization of regulatory RNAs. The majority of the human genome is transcribed, however, less than 1.5% encodes protein. A vast amount appears to be biologically active, non-coding RNAs which are often referred to as the “dark matter” of the cell (Mattick, 2005). Progressively more advanced RNA sequencing techniques have uncovered many classes of small regulatory RNA, however, there is general recognition of three main types: microRNAs (miRNAs), short interfering RNAs (siRNAs) and piwi-interacting RNAs (piRNAs). The full range of these RNA species has been reviewed elsewhere (Carthew and Sontheimer, 2009; Kapranov and St Laurent, 2012). The aim of this article is to focus on miRNAs and their emerging role in neurodegeneration.

Traditionally conditions such as Parkinson's disease, Alzheimer's disease and amyotrophic lateral sclerosis (ALS) have been considered as distinct entities, however, there is increasing evidence of clinical, pathological and genetic overlap. Neurodegenerative diseases can therefore be considered a spectrum of aetiologies culminating in a final common final pathway of neuronal cell death. The pathogenic mechanisms underlying neurodegeneration are complex, but the universal risk factor is aging and there are common themes across the disorders, including protein aggregation, neuroinflammation and mitochondrial dysfunction. There are also common challenges across these conditions including the lack of early diagnostic testing and a large proportion of patients having sporadic forms of the disease (excepting Huntington's disease). Unraveling the similarities and differences between these conditions, and understanding cell type specific vulnerability, will be key to developing new therapeutic interventions.

## Basic biology of miRNA

MiRNAs are a novel class of small (18–25 nucleotides), non-coding RNA molecules predicted to post-transcriptionally regulate at least half the human transcriptome (Friedman et al., 2009). The discovery, and subsequent characterization, of miRNAs has revealed an intriguing additional level of gene regulation that is fundamental in a diverse range of pathways including development, differentiation and pathological processes. Each miRNA is estimated to regulate around 200 targets, and mRNA transcripts may be regulated by multiple miRNAs (Lewis et al., 2003; Krek et al., 2005; Lim et al., 2005). The miRNA biogenesis pathway is highly conserved, as are many miRNA sequences and their target binding sites, highlighting their importance across evolution (Berezikov et al., 2005; Friedman et al., 2009).

MiRNA genes are encoded either in intergenic regions under control of their own promoter, within the introns of protein coding genes or are exonic, overlapping with coding regions and transcribed by the host promoter (Rodriguez et al., 2004). The majority of miRNAs in humans are transcribed independently and putative promoters for the most of these have been identified (Zhou et al., 2007; Ozsolak et al., 2008). Over 40% of human miRNAs are found in clusters that are co-transcribed as polycistronic transcriptional units (Lee et al., 2002; Griffiths-Jones et al., 2008). Many miRNAs are highly temporally and spatially regulated, either via transcription factors or epigenetic mechanisms including DNA methylation and histone modification (Chuang and Jones, 2007). Overall, the mechanisms that control miRNA expression are similar to those of protein-coding genes with a trend toward regulation by their target mRNAs and double-negative feedback loops (Carthew and Sontheimer, 2009).

## miRNA biogenesis

### Canonical pathway

**Figure 1:**
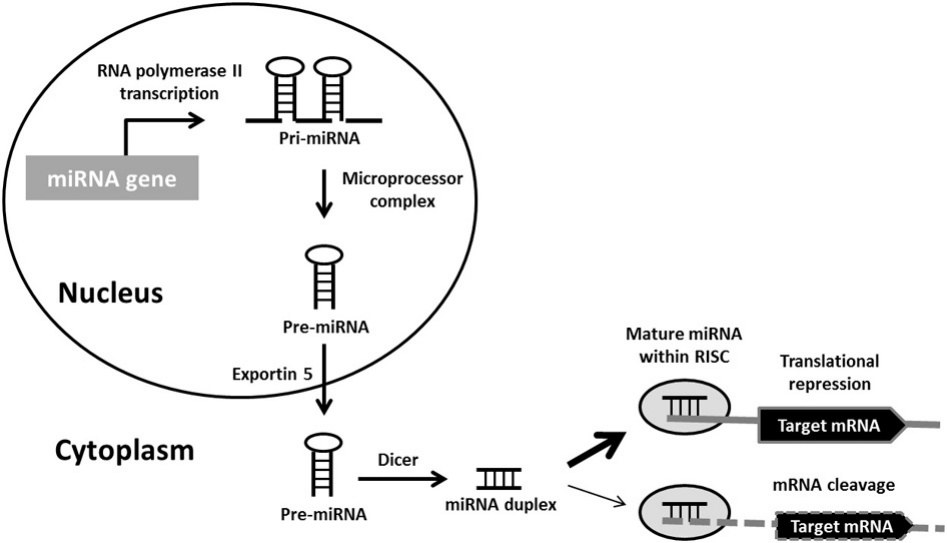
**Canonical miRNA biogenesis pathway.** Primary miRNA (pri-miRNA) transcripts are transcribed by RNA polymerase II. Pri-miRNAs are processed by the microprocessor complex into precursor miRNA (pre-miRNA) hairpins. These are transported into the cytoplasm, where they are further processed by Dicer into miRNA duplexes. Following strand separation, the mature miRNAs are loaded into RNA-induced silencing complexes (RISCs) to guide the repression of protein synthesis or mRNA degradation.

The bulk of miRNAs are generated via the typical, canonical pathway of miRNA biogenesis (Figure 1). MiRNA genes are transcribed by RNA polymerase II (pol II) to generate long primary transcripts (pri-miRNAs), which can be several kilobases long. The pri-miRNAs are capped, spliced and polyadenylated. They may encode a single miRNA, clusters of distinct miRNAs, or a protein and can therefore also act as mRNA precursors (Carthew and Sontheimer, 2009). The next step also takes place in the nucleus and is orchestrated by the microprocessor complex. The principal components of this complex are the RNase III enzyme known as Drosha and its binding partner DiGeorge syndrome critical region gene 8 (DGCR8), a double-stranded RNA-binding protein (Denli et al., 2004). Drosha digests pri-miRNAs to release hairpin structures called precursor miRNAs (pre-miRNAs), which are 60–70 nucleotides in length. Exportin-5 interacts directly with the pre-miRNAs to mediate their export into the cytoplasm, where a second RNase III enzyme named Dicer, cleaves the pre-miRNA to generate a double-stranded miRNA duplex of ~22 nucleotides. Following Dicer processing the miRNA duplex is rapidly unwound as it associates with Argonaute (Ago) proteins, one strand is retained to become the mature miRNA and is loaded into RNA-induced silencing complexes (RISCs) to participate in mRNA regulation. The complementary strand, which is found at lower concentrations within the cell and is sometimes called the * sequence, was believed to be non-functional and rapidly degraded. However, recent studies have demonstrated that several miRNA^*^ sequences associate with different Ago protein complexes to also become active (Czech and Hannon, 2011).

### Non-canonical pathways

The advent of deep-sequencing technologies has led to the discovery of many miRNAs that are generated via alternative mechanisms, by-passing the usual Drosha/Dicer two-step processing (for in depth review see Miyoshi et al., 2010). In mammals four Drosha independent pathways have been identified, namely the mirtron pathway, small nucleolar RNA-derived, tRNA-derived and short hairpin RNA-derived pathways (Babiarz et al., 2008; Ender et al., 2008; Saraiya and Wang, 2008). The most common of these replaces the microprocessor step with a splicing event to produce short hairpin introns known as mirtrons that can be transported by Exportin-5 and cleaved by Dicer (Ruby et al., 2007). Mirtrons are relatively uncommon compared to canonical miRNAs, but have been identified throughout the animal kingdom and there is evidence to suggest a particular importance of mirtrons in the primate nervous system (Berezikov et al., 2007). In addition, there are two Dicer independent miRNA processing pathways. These are very rare with a single miRNA (miR-451) known to be produced via direct pre-miRNA loading onto Ago2 and miRNA-like small RNA sequences generated from tRNAs, with RNaseZ cleavage of pre-miRNAs in place of Dicer (Lee et al., 2009; Cheloufi et al., 2010; Haussecker et al., 2010).

## miRNA mechanism of action

RISC is a generic term for a family of heterogeneous complexes containing Ago proteins that are involved with gene silencing (Pratt and MacRae, 2009). Once incorporated into the RISC, mature miRNAs act as a guide to direct target recognition via base-pairing interactions with mRNA transcripts, which are often located in the 3'UTR region (Bartel, 2009). The majority of animal miRNAs do not match their target sequences exactly, however, nucleotides 2–6 of the miRNA are known as the “seed region” and are critical for target recognition (Lewis et al., 2003, 2005). The extent of complementarity between a miRNA and its target mRNA sequence influences the downstream regulatory mechanism, with perfect matches leading to degradation, while mismatches result in translational repression. In humans the Ago2 protein catalyses target mRNA cleavage and subsequent degeneration of miRNA, although translational repression is the most prevalent mode of action for miRNAs in animals (Liu et al., 2004). The exact mechanism for repression remains unclear. There is evidence to support disruption of translation initiation, promotion of target mRNA deadenylation, sequestration of miRNAs and their targets to processing (P) bodies and stress granules or RISC-mediated protein degradation after translation (Tang et al., 2008). Translational repression by miRNAs is therefore complex and usually produces a fine tuning effect, with a typical miRNA-target interaction producing <2-fold reduction in protein level (Ebert and Sharp, 2012). An additional level of regulation has recently been hypothesized whereby mRNA transcripts compete for common miRNAs by sharing miRNA binding sites. These competing endogenous RNAs (ceRNAs) could be pseudogenes that have the ability to co-regulate gene expression in intricate ceRNA networks (Salmena et al., 2011). Further experimental evidence is, however, required to validate this theory. MiRNAs are recognized as negative regulators of gene expression but there are reports of target activation by miRNAs under certain conditions such as cellular stress (Bhattacharyya et al., 2006; Vasudevan et al., 2007; Orom et al., 2008).

In contrast to miRNA biogenesis, which has been extensively studied and well-defined, the regulation of miRNA degradation and turnover is less clear. MiRNAs are generally considered to be highly stable molecules with a long half-life (Krol et al., 2010b). However, recent studies indicate that miRNA turnover can vary widely among miRNAs and cell types, with rapid miRNA decay a common feature of neuronal cells (Krol et al., 2010a). There is also evidence of miRNA recycling by the cell which may help to explain their capacity to regulate large numbers of transcripts (Baccarini et al., 2011). Mature miRNAs are protected by binding to Ago proteins and the presence of mRNA target sequences are believed to be an important factor in preventing their release from RISC complexes and subsequent degradation (Diederichs and Haber, 2007). Hence, in the absence of complementary mRNA targets, miRNAs could be specifically released to make RISC available for loading new miRNAs. Two families of exonuclease enzymes have so far been identified as mediators of miRNA decay, namely small RNA degrading nuclease (SDN) genes in plants and exoribonuclease 2 (XRN2) in animals (Ramachandran and Chen, 2008; Chatterjee and Grosshans, 2009).

## miRNAs in neurodegenerative disorders

MiRNAs are found in high abundance within the nervous system where they are key regulators of functions such as neurite outgrowth, dendritic spine morphology, neuronal differentiation and synaptic plasticity. The dysfunction of miRNAs in neurodegenerative disorders is increasing recognized, see Table 1 for a summary of the miRNAs discussed within this review.

**Table 1:** **Dysregulated miRNAs discussed within this review article**.

**miRNA**	**Neurodegenerative disease**	**Reported findings**	**Proposed biological effects**	**References**
Let-7	PD	Reduced in two *C.elegans* models of PD (alpha-synuclein transgenic and pdr-1 strains), antagonised by pathogenic LRRK2 in *Drosophila* model	Increased dopaminergic neuronal cell death via increased expression of E2F1 and DP transcription factors	Asikainen et al., 2010; Gehrke et al., 2010
miR-106a^*^	PD	Increased in human PD substantia nigra	Chaperone mediated autophagy pathway	Kim et al., 2007; Alvarez-Erviti et al., 2013
miR-106a/b	AD	Down regulation in human AD temporal cortex and cerebral cortex of APPswe/PSΔE9 AD mouse model	Regulation of APP and ABCA1, impact on Aβ production	Hebert et al., 2008, 2009; Patel et al., 2008; Kim et al., 2012
miR-107	AD	Down regulation in human AD temporal cortex	Regulation of BACE1 and ADAM10, impact on Aβ production	Wang et al., 2008b; Nelson and Wang, 2010; Augustin et al., 2012
miR-124a	AD	Down regulated in human AD temporal cortex	Targets BACE1 and PTBP1, impact on Aβ production	Smith et al., 2011
	ALS	Reduced in spinal cord from SOD1 mouse model of ALS	Regulates glutamate transport protein EAAT2/GLT1	Morel et al., 2013
	HD	Down regulated in human HD frontal cortex and striatum	Down regulated by REST	Marti et al., 2010
miR-125b	HD	Down regulated in *Hdh*^Q111^*IHdh*^Q111^ cell model of HD	Targets HTT, regulates p53 and is predicted to target TBP	Sinha et al., 2010, 2011; Ghose et al., 2011
miR-132	AD	Down regulation in human AD hippocampus, cerebellum and medial frontal gyrus	Regulates several AD associated genes including *SIRT1*, *AChE*, *PTEN*, *FOXO3a* and *p300*	Cogswell et al., 2008; Shaked et al., 2009; Wong et al., 2013
	HD	Down regulated in human HD cortex	Down regulated by REST	Johnson et al., 2008
miR-133b	PD	Down regulated in PD patient midbrain	Negative feedback circuit with Pitx3, function of midbrain dopaminergic neurons	Kim et al., 2007
miR-146a	AD	Up regulated in AD hippocampus and temporal cortex	Targets inflammatory pathways including NFkB and TSPAN12 to potentially impact upon Aβ production	Lukiw et al., 2008; Sethi and Lukiw, 2009; Li et al., 2011
	HD	Down regulated in *Hdh*^Q111^*IHdh*^Q111^ cell model of HD	Targets HTT, TBP and is regulated by the p53 pathway	Sinha et al., 2010, 2011; Ghose et al., 2011
miR-146a^*^	ALS	Up regulated in human spinal cord homogenates	Targets NFL	Campos-Melo et al., 2013
miR-150	HD	Down regulated in *Hdh*^Q111^*IHdh*^Q111^ cell model of HD	Targets HTT regulates p53 and is predicted to target TBP	Sinha et al., 2010, 2011
miR-153	AD	Down regulated in cerebral cortex of APPswe/PSΔE9 AD mouse model	Regulation of APP and APLP2, impact on Aβ production	Liang et al., 2012; Long et al., 2012
	PD	Interaction with SNCA in cell culture models of PD	Targets SNCA	Doxakis, 2010
miR-181c	AD	Down regulated in human temporal cortex and patient serum. Down regulated by addition of Aβ to primary hippocampal neurons	Targets SIRT1, impact on tau metabolism	Hebert et al., 2008; Schonrock et al., 2010, 2012; Geekiyanage et al., 2012
miR-184^*^	PD	Antagonised by pathogenic LRRK2 in *Drosophila* model of PD	Increased dopaminergic neuronal cell death via increased expression of E2F1 and DP transcription factors	Gehrke et al., 2010
miR-200a/c	HD	Up regulated cerebral cortex of N171-82Q HD mouse model	Predicted to target genes involved in neuronal function	Jin et al., 2012
miR-205	PD	Down regulated in PD cerebral cortex tissue	Targets LRRK2	Cho et al., 2013
miR-206	ALS	Up regulation in muscle from ALS patients and ALS mouse model	Nerve-muscle communication and promotes reinnervation following nerve damage	Williams et al., 2009
miR-21^*^	PD	Increased in human PD substantia nigra	Chaperone mediated autophagy pathway	Alvarez-Erviti et al., 2013
miR-224	PD	Increased in human PD substantia nigra and amygdala	Chaperone mediated autophagy pathway, predicted to target lamp-2a mRNA	Kim et al., 2007; Alvarez-Erviti et al., 2013
miR-26b	PD	Increased in human PD substantia nigra	Chaperone mediated autophagy pathway, predicted to target hsc70 mRNA	Kim et al., 2007; Alvarez-Erviti et al., 2013
miR-29a/b	AD	Down regulation in human AD temporal cortex, cerebellum and patient serum	Regulation of BACE1 and impact on Aβ production, regulation of microglia in the aged brain	Hebert et al., 2008; Geekiyanage et al., 2012; Fenn et al., 2013
miR-29c	AD	Up regulation cerebral cortex of APPswe/PSΔE9 AD mouse model	Regulation of BACE1 and impact on Aβ production	Zong et al., 2011
miR-301b	PD	Increased in human PD substantia nigra	Chaperone mediated autophagy pathway	Alvarez-Erviti et al., 2013
miR-338-3p	ALS	Up regulated in human ALS frontal cortex and ALS leukocytes	Predicted to target neurotransmitter signalling pathways	Shioya et al., 2010; De Felice et al., 2012
miR-34a	AD	Up regulated in human AD hippocampus, cerebellum, medial frontal gyrus and white blood cells. Up regulated in cerebral cortex of APPswe/PSΔE9 AD mouse model	Targets BCL2 and potentially increases apoptotic cell death, targets SIRT1, impact on tau metabolism	Schipper et al., 2007; Cogswell et al., 2008; Wang et al., 2009
miR-34b	HD	Up regulated in mutant HTT transfected NT2 cell model of HD and elevated in human HD patient plasma	Interaction with p53 pathway	Gaughwin et al., 2011
miR-34b/c	PD	Down regulation in PD amygdala, frontal cortex and substantia nigra	Altered mitochondrial function and oxidative stress, also linked to brain ageing	Minones-Moyano et al., 2011; Liu et al., 2012
miR-373^*^	PD	Increased in human PD substantia nigra and amygdala	Chaperone mediated autophagy pathway, predicted to target lamp-2a mRNA	Kim et al., 2007; Alvarez-Erviti et al., 2013
miR-433	PD	Polymorphism in *FGF20* binding site linked to increased risk of PD	Increase FGF20 expression and downstream up regulation of SNCA	Wang et al., 2008a
miR-64/65	PD	Reduced in two *C.elegans* model of PD (alpha-synuclein transgenic and cat-1 strains)	Unknown, target candidates include transcription factor mdl-1 and the development gene ptc-1	Asikainen et al., 2010
miR-7	PD	Interaction with SNCA and neuroprotective role in cell culture models of PD	Targets SNCA and supresses SNCA mediated toxicity	Junn et al., 2009; Doxakis, 2010
miR-9	AD	Up and down regulation reported in human AD brain tissue. Down regulated in patient serum and by the addition of Aβ to primary hippocampal neurons	Targets include NFH and SIRT1, plague and tangle formation	Detailed review see Geekiyanage et al., 2012; Schonrock and Gotz, 2012
	ALS	Down regulation in Dicer knock out mature motor neurons and up regulation in SMA mouse model	Targets NFH	Haramati et al., 2010
	HD	Down regulated in human HD cortex	Down regulated by REST and targets REST in a double negative feedback loop	Packer et al., 2008
miR-9^*^	HD	Down regulated in human HD cortex	Down regulated by REST and targets CoREST in a double negative feedback loop	Packer et al., 2008

AD, Alzheimer's disease; PD; Parkinson's disease; ALS, Amyotrophic lateral sclerosis; HD, Huntington's disease.

### Alzheimer's disease

Alzheimer's disease is a complex neurodegenerative disorder and the most common form of dementia in the elderly (Avramopoulos, 2009; Schonrock and Gotz, 2012). The clinical signs of disease are a slow, progressive loss of cognitive function and memory loss, due to destruction of synapses and neurons, which ultimately leads to dementia and death. Alzheimer's disease is progressive with different brain regions and cells affected in a sequential process of increasing deposition of amyloid-β (Aβ) plaques and neurofibrillary tangles of hyperphosphorylated tau as described by Braak staging (Braak and Braak, 1995). Aβ is a mainly 40–42 amino acid fragment derived from the membrane spanning amyloid precursor protein (APP) by proteolytic cleavage by the β-site APP cleaving enzyme (BACE1) and presenilin dependent γ-secretase (Delay et al., 2012).

Less than 1% of Alzheimer's disease cases are familial, with autosomal dominant mutations described in only three genes that lead to early onset disease; *APP*, presenilin 1 *(PSEN1*) and presenilin 2 *(PSEN2)*, both of the latter encoding components of the γ-secretase pathway (Schonrock and Gotz, 2012). No other candidate genes have been identified for familial Alzheimer's disease, although over 500 polymorphisms have been proposed to be risk alleles (Bertram et al., 2007, 2010; Tanzi, 2012). Possession of the ε4 allele of the Apolipoprotein E (*ApoE*) genotype is known to have a modifying influence on the genotype and is associated with a predisposition for the disease. The vast majority of Alzheimer's disease is sporadic, with no obvious genetic component, suggesting that other mechanisms are responsible. Recent studies have demonstrated that alterations in the network of miRNAs contribute to the disease process.

Several studies have used profiling strategies to show miRNA dysregulation in Alzheimer's disease patient brain tissues [see Schonrock and Gotz (2012) for a detailed review of these]. However, little overlap in the specific miRNA changes identified has been observed, which might result from differences in experimental technique, but it is likely that much of this variation derives from differences in the tissue examined and diagnostic features. Comparative miRNA expression in gray and white matter of normal individuals and early stage Alzheimer's disease revealed that most of the disease associated miRNA changes were found in the gray matter. This work highlights that cellular composition of the regions has a marked effect upon the miRNA expression profile, for instance the white matter profile is markedly influenced by the oligodendrocyte content of the tissue (Wang et al., 2011). The use of tissue homogenates, with diverse cell type compositions, and various regions of tissue at different Braak stages, makes comparing the results of individual studies challenging. Therefore, systematic studies investigating the expression of these miRNAs in the different regions of the brain in relation to Braak staging are needed to clarify their significance in relation to the pathogenesis of Alzheimer's disease. However, there are miRNAs that have consistently been identified as dysregulated including miR-107, miR-29, miR-9, miR-181, miR-34, miR-106, and miR-146 (Schonrock and Gotz, 2012). Many of these have been linked to altered regulation of key genes known to be involved with Alzheimer's disease.

Down regulation of miR-107 at an early stage of Alzheimer's disease has been observed in temporal cortex and correlated with the up regulation of BACE1 in two studies, which could impact upon Aβ production (Wang et al., 2008b; Nelson and Wang, 2010) This finding was confirmed as being specific to miR-107 (and not a family member such as miR-103) and demonstrated that as miR-107 declines with advancing pathology, BACE1 increases along with neuritic plaque density (Wang et al., 2008b). Interestingly, miR-107 and miR-124a, two miRNAs experimentally proven to target BACE1 also regulate other aspects of APP metabolism, thus demonstrating the capacity for single miRNAs to influence several components of the same pathway and the potential to produce additive effects. MiR-107 directly targets a disintegrin and metalloproteinase 10 (ADAM10), another secretase enzyme which processes APP, and miR-124a is involved in the regulation of APP mRNA alternative splicing via direct targeting of polypyrimidine tract binding protein 1 (PTBP1) (Smith et al., 2011; Augustin et al., 2012).

The miR-29 family of miRNAs have target sites on BACE1 mRNA and loss of this cluster is negatively correlated with BACE1 expression in a subset of sporadic Alzheimer's disease cases (Hebert et al., 2008; Zong et al., 2011). The correlation was Alzheimer's disease specific and was verified in HEK293 and SH-SY5Y cell culture models, where an increase in Aβ production was also observed as a result. Whilst not specific for brain regions particularly associated with Alzheimer's disease, as demonstrated by analysis of material taken from the cerebellum (a brain area not typically affected by the disease), it is an important additional relationship between an miRNA and mRNA expression (Hebert et al., 2008). In addition to regulating BACE1, miR-29a/b are increased in the aging brain and linked to modulation of microglial activation (Fenn et al., 2013). The miR-29 cluster has been sequenced in a cohort of sporadic and familial patients and variants were found within the cluster that significantly associated with Alzheimer's disease (Bettens et al., 2009). However, this finding requires further validation in additional cohorts and the functional effects of these variants remains unclear.

APP is also a target for miRNA regulation, miR-106a and miR-106b directly bind to APP mRNA and are down regulated in the anterior temporal cortex of Alzheimer's disease patients (Hebert et al., 2008, 2009). Interestingly, miR-106 has also been found to regulate ATP-binding cassette transporter A1 (ABCA1), a lipid transporter implicated in ApoE lipidation and the production of Aβ, suggesting that this miRNA could influence the Aβ generation via more than one route (Kim et al., 2012).

Recent studies have examined possible associations of miR-153 with Alzheimer's disease after functional studies confirmed an interaction with APP and amyloid beta precursor-like protein 2 (APLP2) mRNA transcripts (Liang et al., 2012; Long et al., 2012). Levels of miR-153 were significantly decreased at early and late stages of disease in the APPswe/PSΔE9 double mutant mouse model. Furthermore, the interaction has been demonstrated *in vitro* using HeLa and primary human fetal brain cells where delivery of miR-153 down regulated endogenous expression of APP and APLP2 (Long et al., 2012). miR-153 levels were significantly decreased in the cohort of advanced Alzheimer's disease post-mortem brain specimens with neocortical neurofibrillary tangle pathology (Braak III–VI) as compared with specimens lacking neocortical neurofibrillary tangle pathology (control and Braak stage I/II specimens). Importantly, an inverse co-regulation of miR-153 and APP in human frontal cortex was observed at the protein level (Long et al., 2012). Thus, evidence indicates that miR-153 contributes to post-transcriptional regulation of APP/APLP2 and may therefore have a role in Alzheimer's disease, although further validation of this potential interaction is required.

MiR-9 is a highly conserved, brain enriched miRNA and the most frequently identified misregulated miRNA in Alzheimer's disease to date, although there are inconsistencies regarding up or down regulation as both have been reported (Schonrock and Gotz, 2012). Addition of Aβ to primary neuron cultures results in a rapid decrease of miR-9 *in vitro* and suggests that deregulation may be related to plaque formation (Schonrock et al., 2010). The targets for miR-9 include neurofilament heavy chain (NFH), a protein found in neurofibrillary tangles, and sirtuin (SIRT1), a de-acetylase that interacts with tau and is linked to accumulation of hyperphosphorylated forms of tau in the disease (Haramati et al., 2010; Saunders et al., 2010; Liu et al., 2011; Schonrock et al., 2012). Three other miRNAs have been found to supress SIRT1, namely miR-181c, miR-34, and miR-132, all of which show consistent altered expression in Alzheimer's disease brain (Schonrock and Gotz, 2012; Wong et al., 2013). Furthermore, miR-132 has several direct targets of relevance to Alzheimer's disease pathogenesis including Tensin Homolog (*PTEN*), Forkhead Box O3a (*FOXO3a*), and E1A binding protein p300 (*P300*), which all have a role in neural apoptosis, and the acetylcholinesterase enzyme (AChE), inhibition of which is a standard treatment in Alzheimer's disease and links into the cholinergic anti-inflammatory pathway (Shaked et al., 2009; Wong et al., 2013).

An additional miRNA linked to both inflammation and Alzheimer's disease is miR-146a. This key regulator of innate immunity is up regulated in brain regions affected by Alzheimer's pathology, including the hippocampus and temporal cortex, yet remains unchanged in unaffected regions (Lukiw et al., 2008; Sethi and Lukiw, 2009). Experimentally proven targets of miR-146a include complement factor H (*CFH*), interleukin-1 receptor-associated kinase-1 (*IRAK1*) and TNF receptor-associated factor 6 (*TRAF6*), all associated with innate immunity and inflammatory pathways which are dysregulated in Alzheimer's disease (Wang et al., 2012). Interestingly, miR-146a also targets transmembrane spanning tetraspanin 12 (*TSPAN12*), a key regulator of ADAM10 and therefore has the potential to impact upon Aβ metabolism (Li et al., 2011). These findings further demonstrate the capacity of miRNAs to influence several pathways and mediate cross-talk between pathogenic mechanisms.

### Parkinson's disease

Parkinson's disease is characterized clinically by bradykinesia, tremor and rigidity. This is caused by the progressive loss of dopaminergic neurons in the substantia nigra pars compacta. The majority of cases are idiopathic, however, around 20% of patients have a positive family history. The most important and widely accepted monogenically inherited Parkinson's disease genes are α-synuclein (*SNCA*) and leucine-rich repeat kinase 2 (*LRRK2*) for late-onset disease and Parkin *(PARK2*), oncogene DJ1 (*DJ1*) and PTEN Induced Putative Kinase 1 (*PINK1*) for early onset (Coppede, 2012). The neuropathology of Parkinson's disease is characterized by cellular inclusions known as Lewy bodies in neurons, the main components of which are α-synuclein, neurofilament and ubiquitin.

Recent studies suggest that miRNAs may be involved in the development of Parkinson's disease. Deletion of Dicer in dopaminergic neurons in transgenic mice led to reduced locomotion and symptoms reminiscent of human Parkinson's disease (Kim et al., 2007). Expression profiling of miRNAs from patient midbrain samples revealed a significant decrease in miR-133b. MiR-133b targets Pixt3, a transcription factor enriched in dopaminergic neurons, which is deficient in the aphakia mouse model of Parkinson's disease (Hwang et al., 2003). A negative feedback model has been proposed to explain the relationship, in which, Pitx3 specifically induces transcription of miR-133b and Pitx3 activity is directly down regulated by miR-133b (Kim and Kim, 2007). However, the impact of miR-133b *in vivo* remains unclear, miR-133b null mice display normal midbrain dopaminergic neuronal development and function with a lack of disease phenotype (Heyer et al., 2012).

MiRNA profiling to evaluate dysregulation of miRNAs in various regions of human Parkinson's disease brain tissue has also reported a widespread reduction in the miR-34b/c cluster, which could be detected early in the disease course. Depletion of these miRNAs in dopaminergic neuronal cells led to a reduction of cell viability accompanied by mitochondrial dysfunction (Minones-Moyano et al., 2011). Interestingly, miR-34 has been linked with aging in *Drosophila*, as identified by comparing brain miRNA profiles at three time points, 3, 30, and 60 days. Loss of this age-modulated miRNA in transgenic flies resulted in a late-onset brain degeneration and a striking decline in survival (Liu et al., 2012).

In a recent study Asikainen et al. (2010) used global analysis of miRNAs in three *C.elegans* models of Parkinson's disease. Reduced expression of miR-64 and miR-65 was observed in *SNCA* transgenic and vesicular catecholamine transporter mutant strains, while members of the let-7 family were dysregulated in the *SNCA* and Parkin mutant strains (Asikainen et al., 2010). Let-7 miRNAs are highly conserved and abundant in the central nervous system (CNS) (Lagos-Quintana et al., 2002). Unfortunately there is no literature to describe the function of the miR-64/65 cluster and these results are yet to be validated in rodent models or human tissue.

One of the most important factors in Parkinson's disease pathology is α-synuclein protein accumulation. Mutations and multiplications of the *SNCA* gene are found in familial forms of the disease and polymorphisms in the gene are linked to greater susceptibility in sporadic cases (Hardy et al., 2009). Examination of the *SNCA* gene has revealed an unusually highly conserved and long 3′UTR sequence which is important in the post-translational control of the gene and strongly suggests a role for miRNA regulation (Sotiriou et al., 2009). Two miRNAs have been identified to date as directly targeting *SNCA*, namely miR-7 and miR-153. These brain enriched miRNAs have been found to bind directly to *SNCA* mRNA and down regulate expression, with an additive effect (Doxakis, 2010). In addition, miR-7 suppresses *SNCA* mediated cytotoxicity in neuronal cell models (Junn et al., 2009). Other miRNAs found to be significantly increased in Parkinson's disease brain tissue include six (miR-21^*^, miR-224, miR-373^*^, miR-26b, miR-106a^*^, and miR-301b) that target components of the chaperone-mediated autophagy pathway (Alvarez-Erviti et al., 2013). Defects in this pathway have the potential to disrupt α-synuclein protein degradation and have been proposed as a mechanism for Lewy body pathology (Winslow and Rubinsztein, 2011).

Mutations in the *LRRK2* gene are the most common cause of Parkinson's disease identified to date, but the pathogenic mechanism remains unclear. The LRRK2 protein has been found to directly associate with components of the miRNA processing pathway, including Ago proteins (Dachsel et al., 2007; Gehrke et al., 2010). Pathogenic LRRK2 in *Drosophila* antagonises at least two miRNAs, let-7 and miR-184^*^, leading to greater dopaminergic neuronal cell death via increased expression of E2F1 and DP transcription factors (Gehrke et al., 2010). The pathogenic effects of *LRRK2* mutations were age-dependent. However, this mechanism has yet to be investigated in vertebrate systems and awaits confirmation in human patient tissue models such as *LRRK2* mutant fibroblasts or induced pluripotent stem cell-derived neurons. Interrogation of the *LRRK2* gene sequence has revealed a highly conserved binding site for miR-205 in the 3'UTR. In human and mouse brain tissue the level of miR-205 inversely correlated with LRRK2 protein. Further investigation in Parkinson's disease cases revealed a significant decrease of miR-205 in the frontal cortex compared to controls and *in vitro* luciferase assays confirmed a direct interaction of this miRNA with LRRK2 mRNA (Cho et al., 2013). This novel regulatory mechanism for LRRK2 suggests miR-205 may serve as a therapeutic target for Parkinson's disease.

Another gene associated with increased risk of Parkinson's disease in some populations is fibroblast growth factor 20 (*FGF20*) (Itoh and Ohta, 2013). One polymorphism (rs12720208) is predicted to disrupt the binding site for miR-433 in the 3'UTR of the gene, leading to increased expression of FGF20 and a downstream up regulation of *SNCA* (Wang et al., 2008a). An additional miR-433 putative binding site polymorphism has also been identified in the *SNCA* 3′UTR, however, no difference in allele distribution between patients and controls has been found, and a regulatory effect for miR-433 on *SNCA* expression could not be confirmed (Schmitt et al., 2012).

### Amyotrophic lateral sclerosis

ALS is characterized by the progressive loss of upper and lower motor neurons from the motor cortex, brain stem and spinal cord. For the patient, this results in severe muscle atrophy leading to paralysis and death usually within 2–5 years of symptom onset (McDermott and Shaw, 2008). A family history of ALS is found in 5% of patients, with the remaining 95% of cases sporadic in nature. Clinically, familial and sporadic ALS are very similar, with the exception of an earlier than the typical mid-life onset in some familial cases. Several genes have now been identified as causative in ALS of which the most frequent are *C9ORF72*, superoxide dismutase 1 (*SOD1*), transactive response DNA-binding protein (*TARDBP*) and fused in sarcoma (*FUS*) (Goodall et al., 2012). The proteins encoded by the latter 3 genes, SOD1, TDP-43, and FUS, have been found within the ubiquitinated inclusions that are pathological hallmarks of ALS (Al-Chalabi et al., 2012).

To determine if miRNAs are essential to motor neuron survival, Haramati et al. (2010) used Dicer knockdown to generate transgenic mice lacking the ability to produce mature miRNAs in a subset of their post mitotic motor neurons. The transgenic animals showed progressive locomotor defects and denervation muscle atrophy caused by motor neuron loss. Further work revealed a specific increase in NFH expression, which was at least in part attributed to the loss of miR-9. This is a miRNA highly expressed in the brain and found to be up regulated in mouse models of the juvenile motor neuron disorder known as spinal muscular atrophy (SMA) (Haramati et al., 2010). In addition, miRNAs that directly target neurofilament light chain (NFL) have been found to be altered in ALS. Up regulation of miR-146a^*^ and down regulation of miRNAs 524-5p and 582-3p were reported in SALS spinal cord compared to controls. However, the study used whole spinal cord tissue homogenates so the contribution of differing cell type composition between cases and controls may have influenced the miRNA expression profile differences (Campos-Melo et al., 2013).

The ALS associated proteins TDP-43 and FUS have been found to directly bind key components of the miRNA processing pathway, implicating miRNA dysregulation in disease pathogenesis. Drosha forms two distinct protein complexes, one with DGCR8 which is responsible for the bulk of miRNA processing in the cell (the microprocessor) and a larger complex of at least 17 polypeptides, including TDP-43 and FUS, with limited pri-miRNA processing activity (Gregory et al., 2004). In addition, TDP-43 can directly bind Dicer, Ago2, subsets of pri-miRNAs in the nucleus and pre-miRNAs in the cytoplasm (Kawahara and Mieda-Sato, 2012). Depletion of TDP-43 and FUS protein *in vitro* affects the generation of specific subsets of miRNAs, some of which are implicated in neuromuscular development, neuronal function and survival (Buratti et al., 2010; Kawahara and Mieda-Sato, 2012; Morlando et al., 2012). The mislocalisation of TDP-43 and FUS to cytoplasmic inclusions in ALS is therefore likely to reduce their availability to bind miRNA processing components and affect the production of at least a subset of miRNAs, the consequences of which for neuronal cells have yet to be investigated.

Changes in miRNAs have also been seen in peripheral ALS tissues. Williams et al. (2009) profiled the miRNAs present in the muscle from mutant SOD1 mouse models of ALS. A dramatic increase in the miR-206 was observed in transgenic mice at the time of symptom onset and was found to be a direct result of denervation (Williams et al., 2009). miR-206 is a skeletal muscle enriched miRNA that has fundamental roles in muscle development and plasticity (McCarthy, 2008). A similar increase in miR-206 has also been observed in human ALS patient muscle tissue (Russell et al., 2012). The loss of miR-206 from transgenic SOD1 mice accelerated the rate of disease progression, most likely because miR-206 is a key player in nerve-muscle communication and therefore essential for reinnervation following nerve damage (Williams et al., 2009).

The role of miRNAs as mediators of intercellular communication via exosomes has also been observed in the CNS. Exosomes are small membrane bound vesicles secreted by a variety of cell types including astrocytes and neurons (Raposo and Stoorvogel, 2013). There is evidence that neuronal miRNAs packaged in exosomes can be internalized by astrocytes where they influence protein expression (Morel et al., 2013). Interestingly, this mechanism of regulation has been observed for the main CNS glutamate transporter EAAT2/GLT1. Defects in glutamate transport are well-documented in ALS and a specific decrease in EAAT2/GLT1 levels has been observed in ALS patient samples and the SOD1 mouse model, though the cause of this defect remains elusive (Robberecht and Philips, 2013). Recent work has shown that miR-124a from neuronal exosomes is internalized by astrocytes to result in specific increased expression of EAAT2/GLT1 protein levels via an indirect mechanism. Levels of miR-124a in the spinal cord of mutant SOD1 mouse models is decreased at the end stage of disease and *in vivo* injection of artificial miR-124a oligos into the spinal cord of these mice led to a 30% increase in EAAT2/GLT1 expression. These exciting findings open up the potential for miRNA mediated therapy in ALS to combat the excitotoxicity seen in the disease (Morel et al., 2013).

### Huntington's disease

Huntington's disease is an autosomal dominant inherited disorder caused by an elongated CAG repeat expansion in the huntingtin (*HTT*) gene. The classical motor symptom of chorea is not present in all patients, whilst other motor features such as impaired balance or abnormal fine finger movements are more likely to interfere with the patient's quality of life. Huntington's disease patients frequently develop neuropsychiatric complications such as progressive cognitive decline, personality change and depression. Pathologically, there is severe degeneration of the corpus striatum and atrophy of several brain regions, including the caudate nucleus, putamen and globus pallidus, but also the cortex itself (Zuccato et al., 2010). Medium spiny neurons of the striatum are particularly vulnerable to the *HTT* mutation, which is believed to predominantly cause a toxic gain of function. Although *HTT* is ubiquitously expressed, the aggregates of mutant HTT protein, which are a pathological hallmark of the disease, are restricted to neuronal cells (Imarisio et al., 2008).

There are widespread gene expression changes in Huntington's disease and evidence suggests these can be attributed partly to miRNA dysregulation (Seredenina and Luthi-Carter, 2012). The HTT protein directly interacts with Ago2 and is found to localize to P bodies. Depletion of wild type HTT compromises miRNA mediated gene silencing and the mutant protein disrupts neuronal P body integrity (Savas et al., 2008). There is also evidence to suggest other key components of miRNA biogenesis are dysregulated in mouse models of the disease, including Dicer, Drosha and Exportin-5, at different stages of the disease course (Lee et al., 2011). However, these findings are yet to be further validated.

An alternative mechanism of aberrant transcriptional regulation in Huntington's disease is increased nuclear localization of RE1-Silencing Transcription Factor (REST). REST is a transcriptional repressor that acts to silence neuronal gene expression in non-neuronal cells. In healthy neurons REST is sequestered in the cytoplasm, but in Huntington's disease there is increased nuclear translocation of REST in neurons leading to increased gene repression, which has a negative effect on survival (Zuccato et al., 2007). In addition to targeting mRNA, REST has been shown to regulate miRNAs, including a neuronal miRNA family containing miR-124a, miR-132, miR-9, and miR-9^*^ (Conaco et al., 2006; Johnson et al., 2008; Marti et al., 2010). MiR-124a and miR-132 are highly expressed in the CNS and are crucial regulators of neural identity and function (Conaco et al., 2006; Wanet et al., 2012). Further investigation into miR-9/miR-9^*^ has revealed that they directly target two components of the REST complex to form a double negative feedback network (Packer et al., 2008). The majority of REST-regulated miRNAs identified to date have displayed reduced expression in Huntington's patient brain tissue and models of the disease (Johnson et al., 2008; Packer et al., 2008).

Studies to profile miRNA expression in human tissue, mouse models of disease and cellular systems have revealed numerous expression changes in miRNAs not under REST control, suggesting that miRNA dysregulation is extensive in Huntington's disease (Marti et al., 2010; Sinha et al., 2010; Ghose et al., 2011; Jin et al., 2012). More specifically, the miR-200 family is altered in the cortex of mutant HTT mouse models at early stages of disease, which may compromise a network of genes involved in neuronal plasticity and survival (Jin et al., 2012). In cellular models of Huntington's disease, miR-146a, miR-125b, and miR-150 are down regulated while miR-34b was elevated by the presence of mutant HTT protein (Sinha et al., 2010; Gaughwin et al., 2011). Further investigation revealed complex interplay between these miRNAs and several transcriptions factors, including p53, RelA, and NFkB, (Gaughwin et al., 2011; Ghose et al., 2011). Interestingly, miR-146a, miR-150, and miR-125b also targeted HTT and were predicted to interact with tata binding protein (TBP), a protein known to be recruited into mutant HTT aggregates and were shown to modulate aggregate formation (Sinha et al., 2010, 2011). The relevance of this observation in relation to the pathogenesis of Huntington's disease remains unknown and represents an interesting subject for further investigation (Sinha et al., 2011).

## Clinical applications of miRNA

### Biomarkers

There is an urgent need for effective biomarkers in neurodegenerative disease. For the majority of these conditions, diagnosis relies upon clinical assessment and monitoring the progression of symptoms, which causes substantial delay. Once a neurodegenerative disease has manifested, significant neuronal loss and CNS damage will already be present, therefore early diagnosis is essential to maximize the effectiveness of disease modifying therapies. In addition, neurodegeneration is clinically heterogeneous, with multiple subtypes associated with different survival times, rates of progression and symptoms. Robust biomarkers would be valuable not only for the initial diagnosis, but the classification of various subtypes of disease, monitoring responses to therapeutic agents and tracking disease progression (Shi et al., 2009).

Recent studies have demonstrated the existence of miRNAs in the body fluids including blood, cerebrospinal fluid (CSF) and saliva, at detectable levels where they are exceptionally stable and potential candidates for biomarker discovery (Chen et al., 2008). These extracellular miRNAs are proposed to originate from passive leakage from damaged tissue as the result of cell lysis or apoptosis, active transport from cells via microvesicles such as exosomes or bound within RISC protein complexes (Etheridge et al., 2011).

Blood is an attractive source of biomarkers as it interacts with every tissue in the body and sample collection is already part of standard clinical practice. There has therefore been a recent focus on circulating miRNAs as biomarkers, both extracellular and those expressed in white blood cells, with a number of studies investigating these in neurodegenerative disease patients.

Several studies have interrogated blood-based miRNAs in Parkinson's disease (Margis and Rieder, 2011; Martins et al., 2011; Khoo et al., 2012; Cardo et al., 2013; Soreq et al., 2013). The first determined miRNA expression profiles of peripheral blood mononuclear cells from 19 patients and 13 controls using Exiqon miRCURY LNA assays and identified a panel of 18 significantly dysregulated miRNAs. These were all under-expressed and could differentiate patients from healthy controls (Martins et al., 2011). In order to place these miRNAs in a wider biological context, the authors performed pathway analysis of the predicted target genes of these miRNAs and revealed an over-representation in pathways previously linked to Parkinson's disease, including semaphorin signaling in neurons and transcriptional repression signaling (Martins et al., 2011). A second study investigated 85 miRNAs in whole blood samples using real-time PCR assays from 8 patients to reveal a set of three miRNAs, miR-1, miR-22^*^, and miR-29a, with reduced expression when compared to 8 control subjects. A second set of miRNAs, miR-16-2^*^, miR-26a-2^*^, and miR-30a, was identified as increased in response to levodopa treatment, suggesting a role for anti-parkinsonian drugs in altering miRNA expression (Margis and Rieder, 2011). Two studies have interrogated Parkinson's disease patient plasma samples, Cardo et al. profiled 384 miRNAs from 31 patients at onset of symptoms and 25 controls were compared using TaqMan real-time PCR assays. The study revealed only one significantly up regulated miRNA, namely miR-331-5p, in Parkinson's disease cases (Cardo et al., 2013). Khoo et al. used Agilent microarrays followed by TaqMan QPCR validation to identify a panel of plasma biomarkers for Parkinson's disease consisting of miR-1826, miR-450b-3p, miR-626, and miR-505, which provided 91% sensitivity and 100% specificity (Khoo et al., 2012). Lastly, circulating miRNAs have been profiled in Parkinson's disease patients before and after deep brain stimulation treatment. This study compared leukocyte miRNA expression profiles using SOLiD sequencing in 7 patients before treatment and 6 healthy controls to reveal 16 dysregulated miRNAs, including miR-16, miR-20a, and miR-320. Interestingly, following deep brain stimulation 5 of the 11 leukocyte miRNAs that were significantly altered matched those changed by disease but in the opposite direction (Soreq et al., 2013).

Overall, there is a lack of overlap between these studies and little concordance with the findings from miRNA profiling in CNS tissue in Parkinson's disease, which highlights the difficulties of analysing different sample types and comparing different methodologies.

In ALS, a study of 8 patients and 12 healthy controls revealed 8 miRNAs with significantly altered expression in leukocytes (De Felice et al., 2012). One of these, miR-338-3p is predicted to target genes involved in neurotransmitter signaling pathways and had previously been described as up regulated ALS patient brain tissue, a finding that failed to validate in an enlarged study population and awaits further experimentation (Shioya et al., 2010; De Felice et al., 2012). MiRNA profiling of peripheral monocytes in the SOD1 mouse model of ALS and in ALS patients showed a pro-inflammatory phenotype with high expression of miR-27a, miR-155, miR-146a, and 532-3p in sporadic ALS patients and not in healthy control or multiple sclerosis subjects (Butovsky et al., 2012). A similar profile was also observed in 4 familial ALS patients with *SOD1* mutations, which may represent a common abnormality in the immune system of different forms of ALS (Butovsky et al., 2012).

Huntington's disease is an inherited disorder and can therefore be diagnosed using genetic testing, however, biomarkers are still required for the pre-symptomatic period as this coincides with an opportunity for therapeutic interventions and biomarkers are needed to track disease progression. Circulating levels of miR-34b have been observed at the pre-clinical stage in a small study of Huntington's disease plasma samples when compared to healthy controls. Moreover, miR-34b is induced by the expression of mutant *HTT* gene in neuronally differentiated cell lines (Gaughwin et al., 2011). The study proposes the use of miR-34b as a biomarker for the onset of Huntington's disease, however, the cohort size was small and this findings has yet to be replicated.

In Alzheimer's disease the levels of disease associated miRNAs miR-29a/b, miR-181c, and miR-9 have been reported as down regulated in patient serum samples compared to healthy controls (Geekiyanage et al., 2012). However, the study was conducted in a small study cohort of 7 per group and further validation is required. CSF miRNA signatures have been investigated in Alzheimer's disease patients. Cogswell et al. (2008) recovered miRNAs from CSF samples from 10 Braak stage V Alzheimer's disease patients and 10 Braak stage I patients. Sixty miRNAs were significantly differentially regulated between the different Braak stages, including Let-7 family members, a finding which has since been replicated (Cogswell et al., 2008; Lehmann et al., 2012). Interestingly, extracellular let-7 was shown to activate the RNA-sensing Toll-like receptor (TLR) 7 to mediate neurodegeneration, demonstrating a role for miRNAs as signaling molecules, a function that is independent of their conventional role in gene regulation (Lehmann et al., 2012). In peripheral blood mononuclear cells of Alzheimer's disease patients compared to controls several miRNAs have been identified as differentially expressed including miR-34a and miR-29b, both of which have been found to be dysregulated in brain tissue (Schipper et al., 2007; Villa et al., 2013). Levels of miR-29a were inversely related to SP1, a transcription factor associated with Alzheimer's disease, and is the first reported incidence of a miRNA and its target acting in cooperation as potential biomarkers (Villa et al., 2013). A direct interaction between them, however, remains untested.

Investigation of miRNA-based biomarkers in neurodegenerative disease is in its infancy and has thus far been confounded by small sample sizes, lack of replication and a wide range of methodologies for extraction and quantification of miRNAs. To fully investigate miRNA potential as biomarkers, improved study design, including longitudinal experiments at various disease stages, and standardization of sample preparation and detection methods is required. However, these early studies have highlighted the potential of using circulating biomarkers to measure the effects of disease modifying treatments, an attractive prospect for future neurodegenerative disease clinical trials.

### Therapy

A further clinical application for miRNA is the development of miRNA-based therapy and there are currently several clinical trials testing the therapeutic efficacy of miRNA modulation in other disease areas, such as cancer and chronic hepatitis C viral infection, with more expected with the next few years (Elmen et al., 2008; Nana-Sinkam and Croce, 2013). The therapeutic application of miRNAs can be summarized by two broad strategies, RNA interference (RNAi) using miRNA mimics and miRNA inhibition via miRNA antagonists (including antimiRNA oligonucleotides and sponges).

The use of RNAi techniques to target disease-associated genes, such as *BACE1*, *APP*, *HTT*, *SOD1*, and *SNCA*, holds great promise for neurodegeneration, with a number of studies demonstrating beneficial effects in animal models (Gonzalez-Alegre, 2007; Ling et al., 2011). RNAi faces the same challenges as traditional drug development, including pharmacokinetics, target specificity, efficacy and toxicity (Nana-Sinkam and Croce, 2013). MiRNA strategies are likely to be less toxic, given that they mimic naturally occurring RNAi mechanisms, and there is evidence of reduced immune activation compared to other short hairpin RNAs (shRNAs) when used to treat neurodegenerative disease. McBride et al. (2008) screened several shRNAs that targeted HTT in mouse models of Huntington's disease and found unexpected neurotoxicity caused by microglial activation and astrogliosis. Toxicity was notably reduced when shRNAs were placed into artificial miRNA expression systems (McBride et al., 2008).

One of the advantages of miRNAs as therapeutic agents is their ability to influence multiple target genes and pathways. However, this can also be disadvantageous due to potential off-target effects such as secondary and tertiary consequences of modulating complex miRNA networks. Each miRNA can target several hundred mRNAs, thus understanding the effects of unwanted interactions between the miRNA and endogenous RNAs are important. Another consideration is that artificially introduced miRNAs could overwhelm the biogenesis machinery and impair the effectiveness of endogenous miRNAs (Khan et al., 2009). These saturation-based effects may be of particular importance for neurodegeneration given the evidence of impaired microprocessor function in these conditions. One potential strategy to minimize saturation is to employ non-canonical miRNAs, such as mirtrons, which bypass the microprocessor complex. Proof of principle has been demonstrated by work in Parkinson's disease, where RNAi sequences to *LRRK2* and *SNCA* were incorporated into the miR-1224 mirtron backbone. The artificial mirtron mimics could directly silence human *LRRK2* and *SNCA* in a cell-type specific manner, by using human synapsin promoter in the neuronal SH-SY5Y cell line (Sibley et al., 2012). Artificial mirtrons are therefore an attractive approach for the future, however, their efficacy *in vivo* is yet to be tested.

MiRNA antagonists have also been investigated in models of neurodegeneration. In the ALS SOD1 mouse model, oligonucleotide-based miRNA inhibitors (anti-miRs) to miR-155 have been used to prolong survival and disease duration by 38%. miR-155 was previously identified as up regulated in SOD1 mouse and human ALS patients spinal cord tissues, in addition to patient peripheral blood cells, and is linked to altered inflammation in the disease (Butovsky et al., 2012; Koval et al., 2013). Another experimental strategy to inhibit miRNA function is miRNA sponges, which are based upon competing endogenous RNAs (Ebert and Sharp, 2010). Sponge RNAs contain complementary binding sites to a miRNA of interest and specifically hamper the activity of miRNAs with a common seed sequence. These have yet to be tested for therapeutic applications and have thus far remained in experimental settings.

Effective treatment of neurodegenerative disorders will most likely require manipulation of multiple targets and biochemical pathways. The capacity of miRNAs to modify multiple targets is an attractive feature for developing therapeutic strategies in the future.

## Concluding remarks

**Figure 2:**
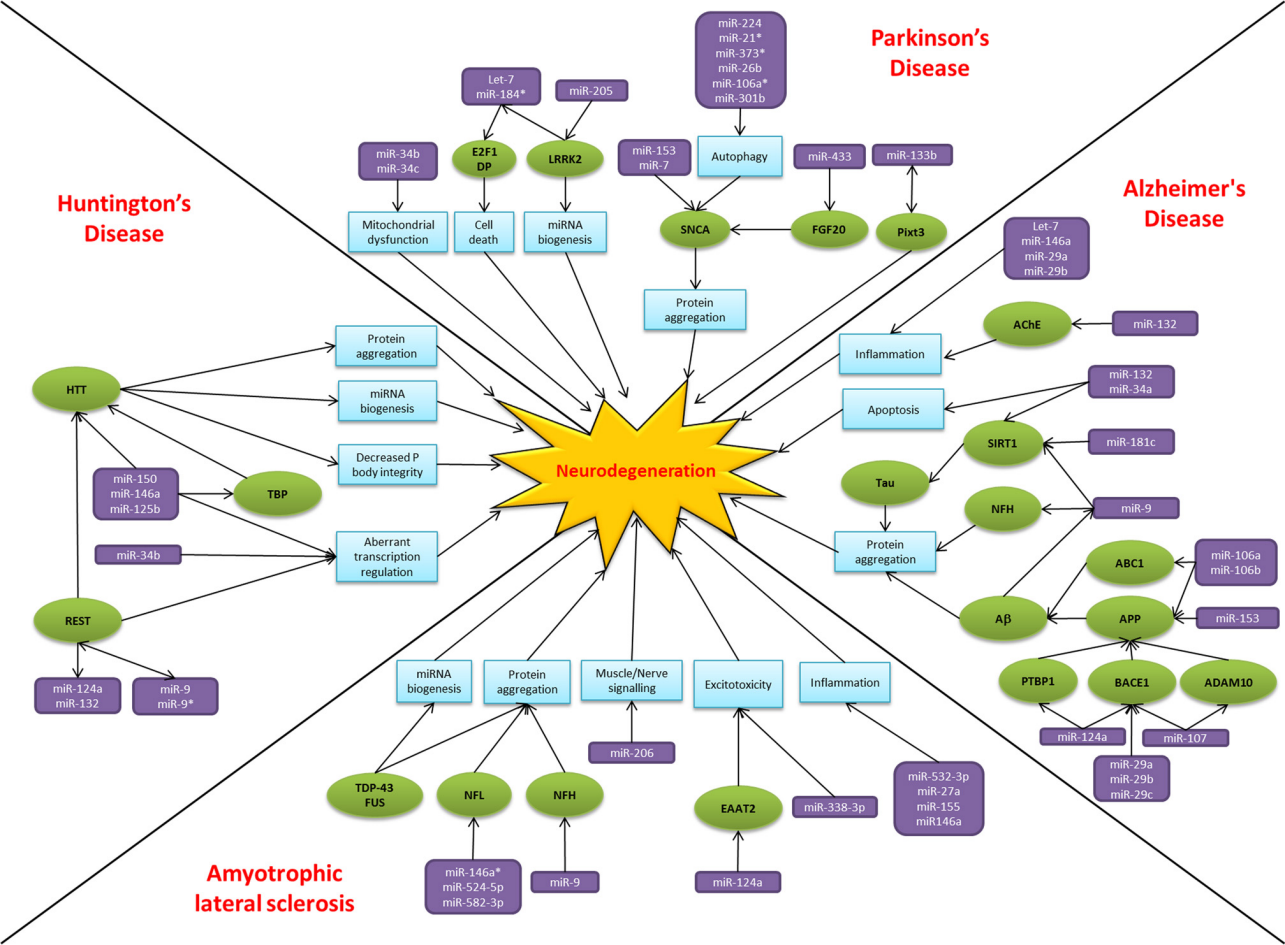
**MiRNAs implicated in neurodegeneration.** A diagram to summarize the miRNA dysfunction networks in Alzheimer's disease, Parkinson's disease, amyotrophic lateral sclerosis and Huntington's disease. Blue boxes indicate pathogenic processes, green circles are affected genes/proteins and the miRNAs are in purple boxes. Arrows indicate the direction of the interactions, culminating in the common pathway of neurodegeneration.

Over the past two decades there has been an explosion of research focused on small non-coding RNAs, the so called “dark matter” of the cell. MiRNAs have emerged as key players in regulating gene expression and their dysregulation is common to many disease states, including neurodegeneration. The alteration of miRNA-mediated regulatory activity potentially upsets the delicate balance required for neuronal cell survival, thereby contributing to pathogenesis and disease progression (Figure 2). In common with proposed disease mechanisms and pathological features, overlap in the dysregulated miRNAs between neurodegenerative conditions are beginning to emerge. Examples of miRNAs with perhaps a more general role in neurodegeneration include miR-9, miR-132, miR-124a, and miR-34. MiR-9 has been found to be dysregulated in Alzheimer's disease, Huntington's disease and animal models of SMA. Reported targets important in terms of neurodegeneration include NFH, SIRT1, BACE1 and REST. Moreover, miR-9 is regulated by Aβ and REST in complex feedback regulatory mechanisms (Packer et al., 2008; Haramati et al., 2010; Schonrock and Gotz, 2012). MiR-132 has been linked to AKT survival signaling, anti-inflammatory pathways and acetylcholine metabolism. There are reports of down regulation causing neuronal death in cell culture models and reduced expression in Alzheimer's disease and Huntington's disease patient tissue (Cogswell et al., 2008; Johnson et al., 2008; Shaked et al., 2009; Wong et al., 2013). MiR-124a is another miRNA which targets BACE1 and has an additional role in excitotoxicity via regulating the glutamate transporter EAAT2 and is itself affected by the Huntington's disease associated transcription factor REST (Marti et al., 2010; Smith et al., 2011; Morel et al., 2013). Lastly, the miR-34 family target SIRT1 to affect tau metabolism, are decreased in Parkinson's disease patients and show increased expression in the presence of mutant HTT (Wang et al., 2009; Schonrock and Gotz, 2012). Overall it is too early to gain an understanding of the scope for miRNAs across the spectrum of neurodegenerative disease. Nevertheless, it is interesting to note that these four miRNAs have been linked to ageing, a key risk factor for neurodegenerative disease, and neuroinflammation, a common pathogenic mechanism (Soreq and Wolf, 2011; Nissan et al., 2012). However, these miRNAs are also reported as brain enriched or neuron specific and may therefore be affected by publication bias as they are the most frequently investigated in the field to date.

The studies highlighted in this review generally have small sample sizes, and results may reflect individual variability within the cohort rather than true disease specific changes in miRNA expression. In addition, many of the studies have focused on miRNA targets related to already known disease genes, such as *LRRK2* in Parkinson's disease and *BACE1* in Alzheimer's disease. A key feature of miRNAs is their short length, making them ideal candidates for non-biased expression profiling techniques, such as next generation sequencing. Such approaches would also address the concern of publication bias that may have affected the field to date. The challenge of unraveling complex gene regulatory networks calls for large, systematic studies of miRNAs in the CNS and continuous development of robust experimental approaches for studying miRNA function. These will need to take into account the issue of disparate CNS/brain regions with divergent cell type composition. Techniques such as laser capture microdissection and induced pluripotent stem cells, in combination with the increased availability of more sophisticated sequencing technologies, means that we can anticipate larger, non-biased, cell type enriched or specific studies of miRNAs for neurodegeneration in the near future.

While the clinical application of miRNAs as biomarkers and therapies in neurodegeneration is perhaps premature, the rate of discovery is promising. In an era of personalized medicine, the use of miRNA expression signatures to sub-classify neurodegenerative disease, provide markers for therapeutic effectiveness and prognosis prediction, is an attractive prospect. Despite the anticipated off-target effects which cannot be fully predicted, saturation of the miRNA biogenesis pathway and possible immune activation, miRNA-based therapy has shown promise in animal models of neurodegeneration. Considerably more groundwork is needed in terms of functional studies to characterize miRNA targets and identify the most appropriate candidates before their potential in clinic can be realized.

### Conflict of interest statement

The authors declare that the research was conducted in the absence of any commercial or financial relationships that could be construed as a potential conflict of interest.
